# Cocaine in Hay-Fever

**Published:** 1892-09

**Authors:** 


COCAINE IN HAY FEVER.—Regarding the use of cocaine in hay fever, Dr. George F. Keifer, of Lafayette, writes the *Journal* as follows:

The hay fever season is upon us. A common prescription reads thus: Cocaine muriate, ten grains; water, one half ounce. Spray as necessary to control paroxysms.

Cocaine is used thus because it produces relief, and for no other reason. I wish to enter an unqualified objection to any prescription made thus, calling for the use of cocaine *ad libitum*.

Cocaine is an admirable anesthetic. One of its actions is the contraction of mucous membranes with which it comes in contact. For the action of cocaine upon mucous membranes there is the reaction of relaxation of mucous membranes. Let that relaxation be produced repeatedly, and it is soon a chronic relaxation which I have found in a number of instances to be polypoid in character. Bosworth writes that the primary lesion, probably in most cases, is the obstruction, as shown by Harrison Allen. Why make the nasal obstruction worse? The rational treatment is to get rid of that obstruction, according to the general principles of surgical practice. Moreover, I have seen several cases where the patient was made a total wreck by the formation of the cocaine habit.—*Indiana Medical Journal.*

